# Cumulative multisensory discrepancies shape the ventriloquism aftereffect but not the ventriloquism bias

**DOI:** 10.1371/journal.pone.0290461

**Published:** 2023-08-22

**Authors:** Christoph Kayser, Hame Park, Herbert Heuer

**Affiliations:** 1 Department of Cognitive Neuroscience, Universität Bielefeld, Bielefeld, Germany; 2 Department of Neurophysiology & Pathophysiology, University Medical Center Hamburg-Eppendorf, Hamburg, Germany; 3 Leibniz Research Centre for Working Environment and Human Factors, Dortmund, Germany; University of Canberra, AUSTRALIA

## Abstract

Multisensory integration and recalibration are two processes by which perception deals with discrepant signals. Both are often studied in the spatial ventriloquism paradigm. There, integration is probed by the presentation of discrepant audio-visual stimuli, while recalibration manifests as an aftereffect in subsequent judgements of unisensory sounds. Both biases are typically quantified against the degree of audio-visual discrepancy, reflecting the possibility that both may arise from common underlying multisensory principles. We tested a specific prediction of this: that both processes should also scale similarly with the history of multisensory discrepancies, i.e. the sequence of discrepancies in several preceding audio-visual trials. Analyzing data from ten experiments with randomly varying spatial discrepancies we confirmed the expected dependency of each bias on the immediately presented discrepancy. And in line with the aftereffect being a cumulative process, this scaled with the discrepancies presented in at least three preceding audio-visual trials. However, the ventriloquism bias did not depend on this three-trial history of multisensory discrepancies and also did not depend on the aftereffect biases in previous trials ‐ making these two multisensory processes experimentally dissociable. These findings support the notion that the ventriloquism bias and the aftereffect reflect distinct functions, with integration maintaining a stable percept by reducing immediate sensory discrepancies and recalibration maintaining an accurate percept by accounting for consistent discrepancies.

## Introduction

Our brain combines multisensory signals to guide immediate behavior, but discrepant multisensory signals can also exert lasting influences even on subsequent unisensory judgments. A prototypical example is the spatial ventriloquism paradigm: here the discrepant positions of visual and auditory stimuli are combined when localizing the sound ‐ the ventriloquism bias. In addition, both signals can influence the localization of subsequent unisensory auditory stimuli–the ventriloquism aftereffect [1–9]. This aftereffect–or recalibration bias ‐ emerges in the absence of explicit task feedback and on multiple time scales [4, 10]. Importantly, both integration and recalibration are typically described by their dependency on the spatial discrepancy presented in the multisensory trials. In fact, their similar dependency on this multisensory dimension can be taken to suggest that both arise from a common underlying multisensory mechanism.

Indeed, one line of work supports the notion that the aftereffect is a direct consequence of the preceding integration of multisensory signals [11–13]. For example, the discrepancy between integrated multisensory signals and subsequent unisensory stimuli apparently drives recalibration [4–6, 12, 13], and both biases are strongest when the multisensory stimuli are judged as being causally related [6, 14]. Furthermore, both are similarly affected by manipulations of stimulus reliability [15] and attention [7], and neuroimaging studies have pointed to partly overlapping neurophysiological processes that shape integration and recalibration [5, 16]. If this notion were correct, experimental manipulations affecting integration should also affect recalibration. For example, both biases should depend in a similar manner on the history of the multisensory experience, such as the discrepancies between the auditory and visual signals experienced over the last few seconds or minutes.

An alternative view stipulates that recalibration is independent of whether the preceding multisensory signals had been integrated. Rather this view suggests that recalibration is shaped by a belief in a modality-specific bias, that is, a bias that is specific to the auditory modality and hence pertains specifically to estimates of acoustic features and does not require multisensory stimuli to be present [1, 13, 17–21]. This belief in a modality specific bias may be driven by many factors, but also by multisensory evidence, hence the observed scaling of the aftereffect with the degree of multisensory discrepancy. If this view were correct, integration and recalibration could be differentially affected by the recent history of experienced multisensory stimuli. While some previous studies have probed the history dependence of the aftereffect in general, these did not provide a direct comparison of integration and recalibration biases or featured experimental designs that were limited by the use of fixed and predictable spatial discrepancies [1, 6, 9, 10].

To arbitrate between these two views, we analyzed data from ten experiments and directly tested whether the spatial ventriloquism bias and the aftereffect depend in a similar manner on the history of multisensory discrepancies experienced over several preceding trials. All experiments featured a prototypical series of audio-visual and auditory trials (AV-A), which in the original experiments was used to probe the scaling of integration (in the AV trial) and of immediate recalibration (A trial) with the spatial discrepancy presented in the immediate AV trial. Importantly, the direction and magnitude of the multisensory spatial discrepancies was variable and unpredictable from trial to trial, different to other previous studies [1, 6, 9, 10]. We here leveraged this data to model both biases against the sequence of multisensory discrepancies across up to three previous AV trials. Our results provide converging and strong evidence that the aftereffect scales in a cumulative fashion with the series of experienced discrepancies, while multisensory integration in the form of the ventriloquism bias does not.

## Materials and methods

### Experimental design and procedures

We analyzed data from 10 experiments. For eight of these we have previously published some results pertaining to the ventriloquism bias or the aftereffect, but not the specific analyses implemented here (see Table 1 for details). All experiments followed the same overall design template to probe the ventriloquism bias and the immediate aftereffect following established work [5, 6, 14, 16, 22, 23]. Participants were adult volunteers who reported no history of neurological diseases, and normal vision and hearing. They were compensated for their time and provided written or electronic informed consent prior to participation. Before the actual experiments we performed a screening task to probe participants’ spatial hearing thresholds using similar auditory stimuli as used in the main experiments [22]. More precisely, we only included participants who correctly identified lateralized sounds (from the four eccentric speakers of each experiment) on at least 75% of trials in this screening task. The actual performance of the included participants was well above 90% correct for all datasets. The studies were approved by the local ethics committee of Bielefeld University and data were collected anonymously. This makes it impossible to determine whether some participants participated in more than one of the experiments reported here.

**Table 1:** Datasets. The datasets analyzed here come from 10 experiments that capitalize on the same overall task design. Experiments 1–8 have been published as part of previous studies, though the data were not analyzed for the present purpose. Experiments 9 and 10 are analyzed for the first time and contained additional manipulations not analyzed here. N indicates the number of participants. The last three columns indicate the spatial separation of the five stimulus locations, the number of AV-A trial-pairs per participant and the fraction of V trials that were not analyzed here.

Experiment	Publication	Note	N participants	Stimulus spacing	AV-A pairs	V trials
1	[16]	Short-term recalibration data only	19	11.6°	360	7%
2	[14]	Experiment 1	20	8°	375	10%
3	[14]	Experiment 2	21	8°	375	10%
4	[14]	Experiment 3 ‐ mask	22	8°	180	10%
5	[14]	Experiment 3 –no mask	22	8°	180	10%
6	[23]	Young participants only	22	8.5°	396	8%
7	[22]	Narrow context	21	6.7°	396	8%
8	[22]	Wide context	21	11.6°	396	8%
9	Previously unpublished	Trial context manipulation	17	11°	465	12%
10	Previously unpublished	Naturalistic stimuli	20	11°	360	15%

**Fig 1:**
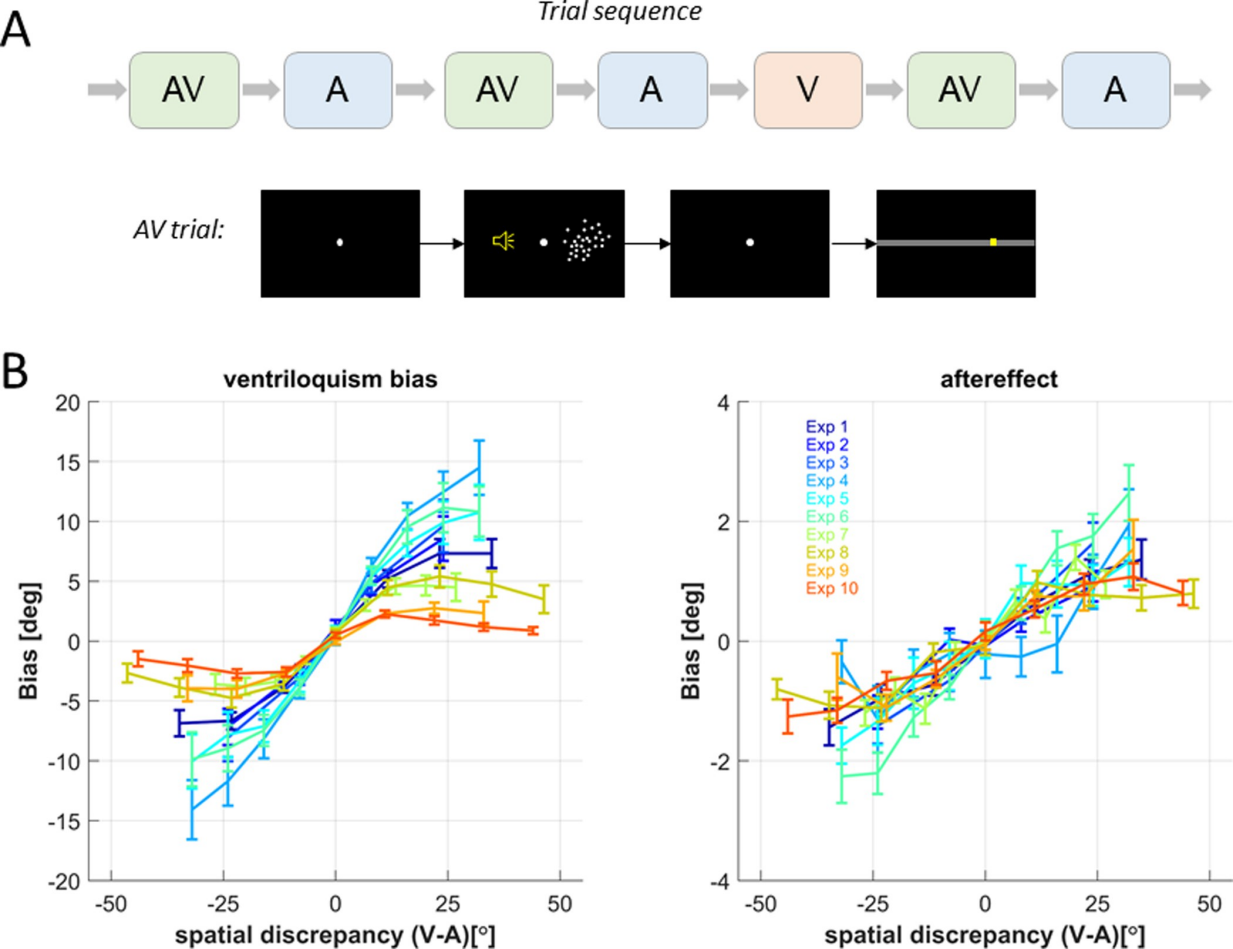
Experimental design and response biases. **A)** shows the typical sequence of AV and A trials designed to probe the two responses biases, and the less frequent V trials to maintain attention on both modalities. The lower panel in A shows a typical sequence of events within an AV trial, including a fixation period, stimulus presentation (here random dots), a post-stimulus period and the response bar on which participants moved a mouse cursor to indicate the perceived location of auditory or visual stimuli. The yellow speaker icon indicates the location of a sound, but was not displayed in the experiment. **B)** shows the average (mean and s.e.m.) bias for each of ten experiments as a function of the spatial discrepancy presented in the AV trial probing the ventriloquism bias or the AV trial immediately prior to the A trial probing the aftereffect.

In all experiments, the auditory and visual stimuli were presented either simultaneously in multisensory trials (AV) or individually in unisensory trials (A or V; c.f. Fig 1A). The experiments were performed in a dark and anechoic chamber with sounds being presented at about an r.m.s level of 65 dB SPL through one of 5 speakers (Monacor MKS-26/SW, MONACOR International GmbH & Co. KG, Bremen, Germany) located at 5 horizontal locations. The precise spacing of stimulus locations differed between experiments but the center speaker was always at 0 azimuth (Table 1). The visual stimuli were projected (Acer Predator Z650, Acer Inc., New Taipei City, Taiwan) onto an acoustically transparent screen placed in front of the speakers (Screen International Modigliani, 2x1 m^2^) and were centered on the same five locations as the sounds. The positions of auditory and visual stimuli were drawn pseudo randomly to sample a large range of multisensory discrepancies (the spatial separation of the auditory and visual stimuli in the AV trials) and stimulus positions. This made the direction of the spatial discrepancy presented in each trial and the magnitude of this unpredictable to the participants, unlike in some other previous studies that used a fixed magnitude or a fixed direction of spatial discrepancy for each participant [1, 9]. The participants were asked to localize the sound (on AV and A trials) or to localize the visual stimulus (on V trials). They responded by moving a mouse cursor to the respective location and clicking to submit their response. The AV trials served to probe the ventriloquism bias and to induce the aftereffect; the A trials served to probe the aftereffect; the less frequent V trials served to ensure that participants maintained divided attention across both senses. The AV trials were always followed by A trials, which were sometimes followed by V trials. The overall number of trials and the proportion of V trials differed between experiments (see Table 1).

#### Stimuli experiments 1–9

The visual stimulus was a cloud of white dots distributed according to a two-dimensional Gaussian distribution (200 dots, SD of vertical and horizontal spread: 1.6° (Experiments 2–5,7,8) or 2° (Experiments 1,6,9), width of a single dot: 0.12°, duration of stimulus presentation: 50 ms), and the auditory stimulus was a 1300 Hz pure tone (50 ms duration, with 8ms on/off cosine ramps). Experiment 9 was originally designed to probe the impact of trial-context and presented the AV, A and V trials in three sub-blocks featuring different proportions of AV trials with a visual stimulus being displaced from the sound towards the center of the visual screen. For the present analysis, however, we combined all trials and ignored this specific manipulation.

#### Stimuli experiment 10

For this experiment we used naturalistic images and sounds to probe whether the effects generalize to naturalistic stimuli. More precisely, the visual stimuli were either the faces of one of five animals (cat, dog, eagle, sheep, tiger) or phase-scrambled versions of these images. The images were 200x200 pixel large. Scrambled images were generated by dividing the original images into 400 10x10 pixel blocks and randomly assigning each block to a random block-position in a random image. For presentation the images were faded into the grey background using a Gaussian mask with a horizontal SD of 2° and were equalized for luminance and contrast. The acoustic stimuli were the matching five vocalizations (with 200 ms duration, all r.m.s normalized) or scrambled versions of these. These scrambled sounds were created by filtering the original sounds into 10 equi-spaced band-limited components between 100Hz and 8kHz. We then randomly assigned these 50 band-limited components to the individual five scrambled conditions and obtained the final scrambled sound as the average of the respectively-assigned 10 components. AV trials either presented matching naturalistic auditory and visual pairs, scrambled pairs, or pairs featuring one naturalistic and one scrambled stimulus (again these specific manipulations are neglected for the present analyses).

### Analysis of response biases

We used identical definitions of the trial-wise ventriloquism bias and the aftereffect to facilitate a direct comparison of these. Each bias was defined as the difference between the reported sound location (in the AV trial for the ventriloquism bias, the A trial for the aftereffect) and the average reported location in all A trials for this specific sound position. This definition ensures that other response tendencies, such as to report stimuli closer to the midline as they are (also known as central bias), or to report them slightly shifted towards either side, are equally accounted for in the ventriloquism bias and the aftereffect [6, 9, 23, 24]. Typically, the trial-wise biases are then investigated in relation to the multisensory discrepancy in the respective AV trial in which the ventriloquism bias is obtained, or the AV trial immediately prior to the A trial in which the ventriloquism aftereffect is obtained (Fig 1B). We here extended this analysis and probed each bias in relation to the discrepancies in up to three successive AV-A trial-pairs. For this analysis we ignored the fact that some AV-A trial-pairs were interspersed with visual only trials. This is reasonable given that these visual stimuli should not affect auditory recalibration and given that the ventriloquism bias is robust against intervening mnemonic manipulations or other unisensory judgements [1, 14].

Both biases scale with the sign and magnitude of the spatial discrepancy (Fig 1B for experiment-wise averages). This dependency can taper off at large discrepancies, reflecting the reduced combination of spatially very discrepant signals. Such a non-linear dependency is predicted by models of multisensory causal inference, which posit that multisensory signals are only combined when they are likely to arise from a common source [13, 25–28]. To account for such nonlinear dependencies, we included both linear and nonlinear predictors in the analyses described below, analogously to our previous work [14, 16, 22, 23]: for a given spatial discrepancy, Δva, the non-linear term was modelled as sign(Δva) * sqrt(Δva). In the model below this is abbreviated as Δva^0.5^.

**Fig 2:**
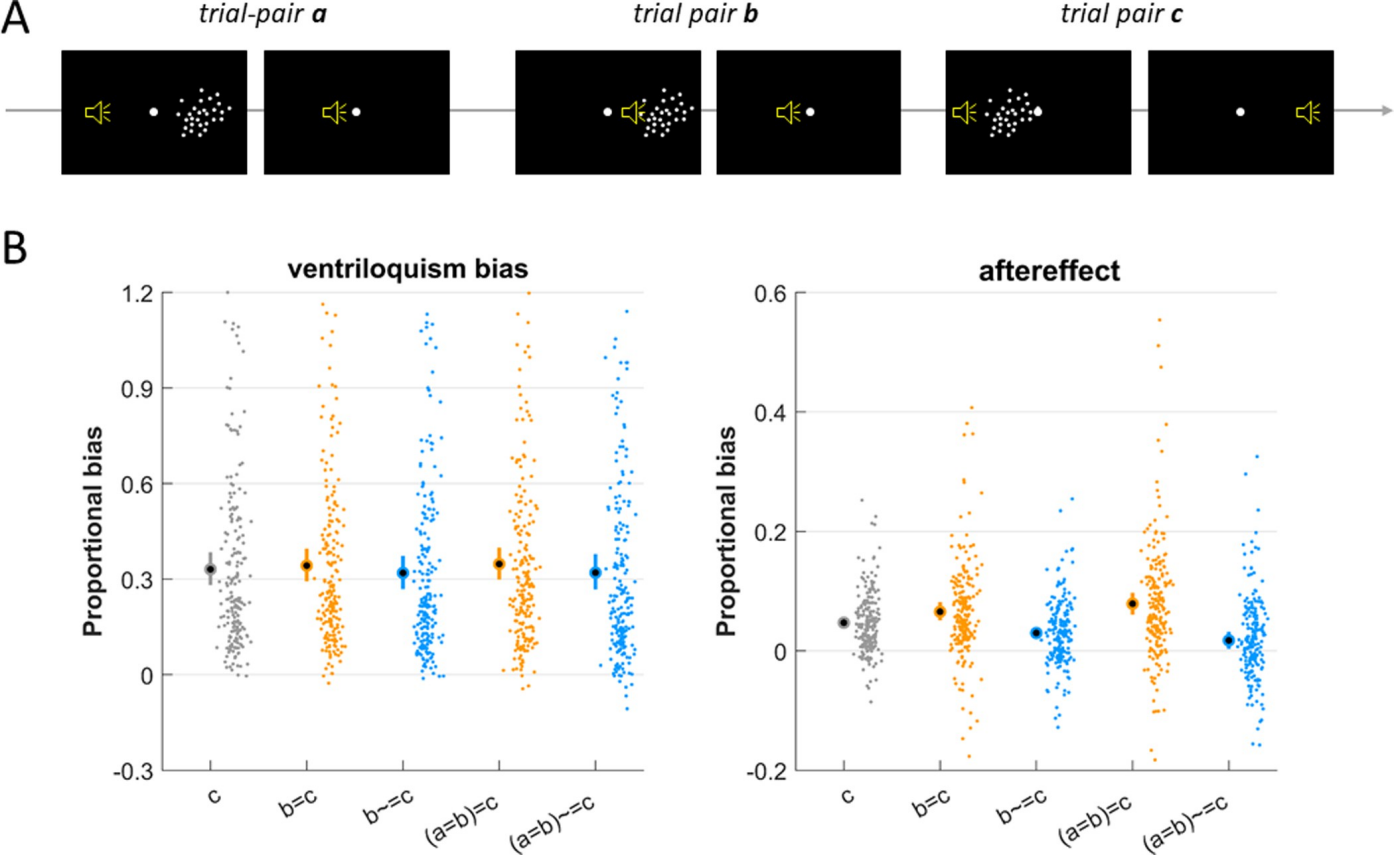
History-dependence is prominent for the aftereffect but not for the ventriloquism bias. **A)** We probed the dependency of each bias obtained in a given AV-A trial-pair (termed c) on the spatial discrepancy in that trial-pair and also two preceding trial pairs (labelled a and b). For the analysis in this figure we grouped trial-pairs based on the direction of the spatial discrepancy, for analyses reported below we included also the precise numeric values. **B)** Proportional biases contingent on the history of the sign of the multisensory discrepancy. The bias was quantified for individual AV-A pairs (condition c), sequences of two pairs with same (‘b = c’) or opposing sign of discrepancy (‘b~ = c’), triplets featuring the same sign (‘a = b = c’) and triplets featuring a change in sign (‘a = b~ = c’). The criterion of an equal sign of the discrepancy selects trials on which the visual stimulus is always on the same side of the auditory stimulus, regardless of their absolute locations. The proportional bias was defined as the ratio of trial-wise bias relative to the discrepancy in trial-pair c. Dots reflect individual datasets (i.e. participants, n = 205), circles the overall mean and error bars the 99% bootstrap confidence interval.

We quantified the dependency of each bias on the spatial discrepancies in up to three successive AV-A trial-pairs, using different analyses that each have their own merits and benefits. In a first analysis, we compared proportional biases contingent on different constellations of the sign of the spatial discrepancy in preceding trial-pairs (Fig 2A). For this we focused on sequences of trial-pairs (denoted by a,b,c in the following) that all featured a non-zero spatial discrepancy. The proportional bias was computed as the ratio of the trial-wise bias observed in pair c relative to the discrepancy in trial-pair c. We derived this for the following constellations of trials: across all trial-pairs (‘c’ in Fig 2B), for sequences of two pairs with same (‘b = c’) or opposing (‘b~ = c’) signs of the discrepancy, and for triplets with the same sign (‘a = b = c’) or a change in sign (‘a = b~ = c’). Including more than three preceding trials was not possible as this would have left insufficiently many trials for the respective constellations of interest. This analysis has the benefit of providing an easy-to-interpret visualization of the history dependence (Fig 2) but has the limitation of not including trials with zero discrepancies and potential nonlinear dependencies on the spatial discrepancy.

In a second analysis, we fit participant-wise linear models to predict each trial-wise bias (in trial-pair c) based on the precise discrepancies in this and up to two preceding trial-pairs. These models included both linear and non-linear predictors for the discrepancies and included also trials with zero discrepancy (in contrast to the above analysis of proportional biases). Specifically, we modelled the trial-wise bias based on the immediate discrepancy (in trial-pair c), by also including one previous pair (b&c) or by including two preceding pairs (a&b&c). We then compared the explained variance (R^2^) between these models across participants. The precise model when including only trial-pair c was as follows, with *bias* being the trial-wise bias in trial-pair c and Δ_c_va the spatial discrepancy in this trial-pair : *bias* ~ 1 + Δ_c_va + Δ_c_va^0.5^ ; models which included more previous trial-pairs were extended by the respective spatial-discrepancy terms for trials b and a. The regression weights and full formulas for these models are reported for the sake of completeness in the S1 File.

In addition, we used variance partitioning to quantify the variance that is uniquely explained by the immediate discrepancy (c) and the preceding discrepancies (a,b). Variance partitioning splits the explained variance into proportions uniquely explained by individual predictors (also known as semi-partial correlations) and those common to two or more predictors [29–31]. In the present data these common variances were mostly negligible. Because fitting linear models with multiple predictors on limited data can result in biased estimates of variance, and because individual experiments contained different numbers of trials, we corrected the obtained variance estimates: for each model we obtained an estimate of the statistical bias (i.e. the variances explained by chance) by fitting the models 500 times after randomizing the dependent variable across trials [32]. The median of these 500 variance estimates was then subtracted from the original estimates. As a result of this bias correction, the corrected variance estimates can be negative, with small values indicating near chance-level results.

In a third analysis, we analyzed the combined data across all ten experiments using single linear mixed effect models. In contrast to the above participant-wise regression models, this analysis also allows for systematic between-experiment differences in the dependencies on spatial discrepancies. We fit models including only the immediate trial-pair (c) or also including the discrepancies of one or two previous trial-pairs. These models again included both linear and non-linear predictors for each discrepancy and included random offsets and slopes for the individual discrepancy predictors for each experiment (see S1 File for formulas). For each bias we compared the predictive power of the three models (including trials c, b&c, a&b&c) using their BIC values [33, 34]. Given the large number of predictors, this analysis may underestimate the relevance of previous trials as models including three trial-pairs may be overly punished for their high number of predictors. Still, we found that this analysis and that using models fit to individual datasets above yield the same overall conclusions.

Because the data in Fig 1 may be taken to suggest that the degree of between-experiment variability in the group-data could be larger for the ventriloquism bias than the aftereffect, we quantified the between-experiment variability in two ways. For this analysis we focused on the proportional bias computed over all trials excluding those with zero discrepancy (hence as shown in Fig 2B, condition c). For each experiment we averaged the proportional bias across participants. Then we computed the coefficient of variation across experiments, defined as the ratio of standard deviation across experiments divided by the mean across these. We also quantified the overall range of between-experiment variability, defined as the difference between the largest experiment-wise proportional bias minus the smallest one, divided by the mean across experiments.

### Statistical analysis

Statistical comparisons are based on the 205 datasets analyzed, which we treated as independent samples, though it is possible that some participants completed more than one experiment. To describe condition-wise effects across the sample we relied on population means and 99^th^ percentile bootstrap confidence intervals obtained using 5000 randomizations [35]. For a statistical assessment of differences between conditions we relied on paired t-tests for which we report Cohens’ D as effect size and Bayes factors (BF) as indicators of significance. Because the present study emphasizes the qualitative and quantitative comparison of the two perceptual biases we refrain from reporting p-values but emphasize effect sizes and Bayesian statistics [36–38]. When interpreting BF’s we refer to the nomenclature of Raftery [34]: we interpreted BF between 1 and 3 as ‘weak’, between 3 and 20 as ‘positive’ and between 20 and 150 as ‘strong’ evidence.

## Results

We analyzed the ventriloquism bias and the immediate aftereffect in 205 datasets that were collected across ten variations of the same overall experimental design (see Table 1). This design consisted of a sequence of alternating audio-visual (AV) and auditory (A) trials, interspersed with visual trials that served to maintain attention divided across modalities (Fig 1A). In this design the AV trials allow quantifying the ventriloquism bias as a function of the multisensory spatial discrepancy presented in that trial. The subsequent A trials allow quantifying the aftereffect as a function of the discrepancy in the immediately preceding AV trial.

Fig 1B shows the experiment-averaged biases as a function of the spatial discrepancy in the respective AV-A trial-pair. These reveal a general increase in bias with increasing discrepancy but also a tapering off at higher discrepancies that is more prominent for some than other datasets. This nonlinear scaling is expected based on models of multisensory causal inference. These predict a stronger bias when two stimuli are judged as originating from a common source and a weaker bias when judged as originating from distinct sources [24, 39]. Given that the spatial separation provides one important cue for a common origin, this gives rise to the nonlinear dependency of the bias on discrepancy.

Comparing the biases between experiments in Fig 1 may suggest that the between-experiment variability for the ventriloquism bias was larger than for the aftereffect. However, this was not the case. The between-experiment coefficient of variation of the proportional biases was comparable for the ventriloquism bias (CV = 0.90) and the aftereffect (CV = 0.89). Similarly, the overall range of between-experiment variation of the proportional biases, when normalized to the respective mean of each bias, was also very comparable (ventriloquism bias 2.74, aftereffect 2.75). This suggests that one can combine the data across experiments similarly to analyze both biases.

Our main question was whether each bias depends only on the spatial discrepancy experienced in the immediate AV-A trial-pair, or whether these also depend on the discrepancies presented in preceding AV trials. In a first analysis we quantified the proportional biases for sequences of trial-pairs that were selected for specific constellations of the direction of audio-visual discrepancies (Fig 2). Specifically, we compared the proportional biases for sequences featuring the same sign of discrepancy (e.g. ‘b = c’, ‘a = b = c’), or for sequences featuring a change in sign (e.g. ‘a~ = b’, ‘a = b~ = c’).

This revealed a cumulative influence of discrepancy on the aftereffect that is only weakly present for the ventriloquism bias. Following sequences of three trial-pairs featuring the same direction of discrepancy the ventriloquism bias was largely comparable to that in sequences featuring a change in sign (‘a = b = c’: 0.348 [0.299 0.401], mean and 99% bootstrap CI, ‘a = b~ = c’: 0.320 [0.268 0.376]); in contrast, the aftereffect differed considerably (‘a = b = c’: 0.079 [0.062 0.098], ‘a = b~ = c’: 0.018 [0.002 0.032]). A direct comparison provided positive evidence for a difference between constellations for the ventriloquism bias with a small effect size (paired t-test ‘a = b~ = c’ vs. ‘a = b = c’: BF = 10.2, Cohens’ D = 0.22), while for the aftereffect the evidence for a difference was very strong and the effect size moderate (BF = 10^7^, Cohens’ D = 0.46). While this analysis clearly visualizes the differences between biases it was restricted to trial sequences featuring only non-zero discrepancies and did not account for potential non-linear dependence.

**Fig 3:**
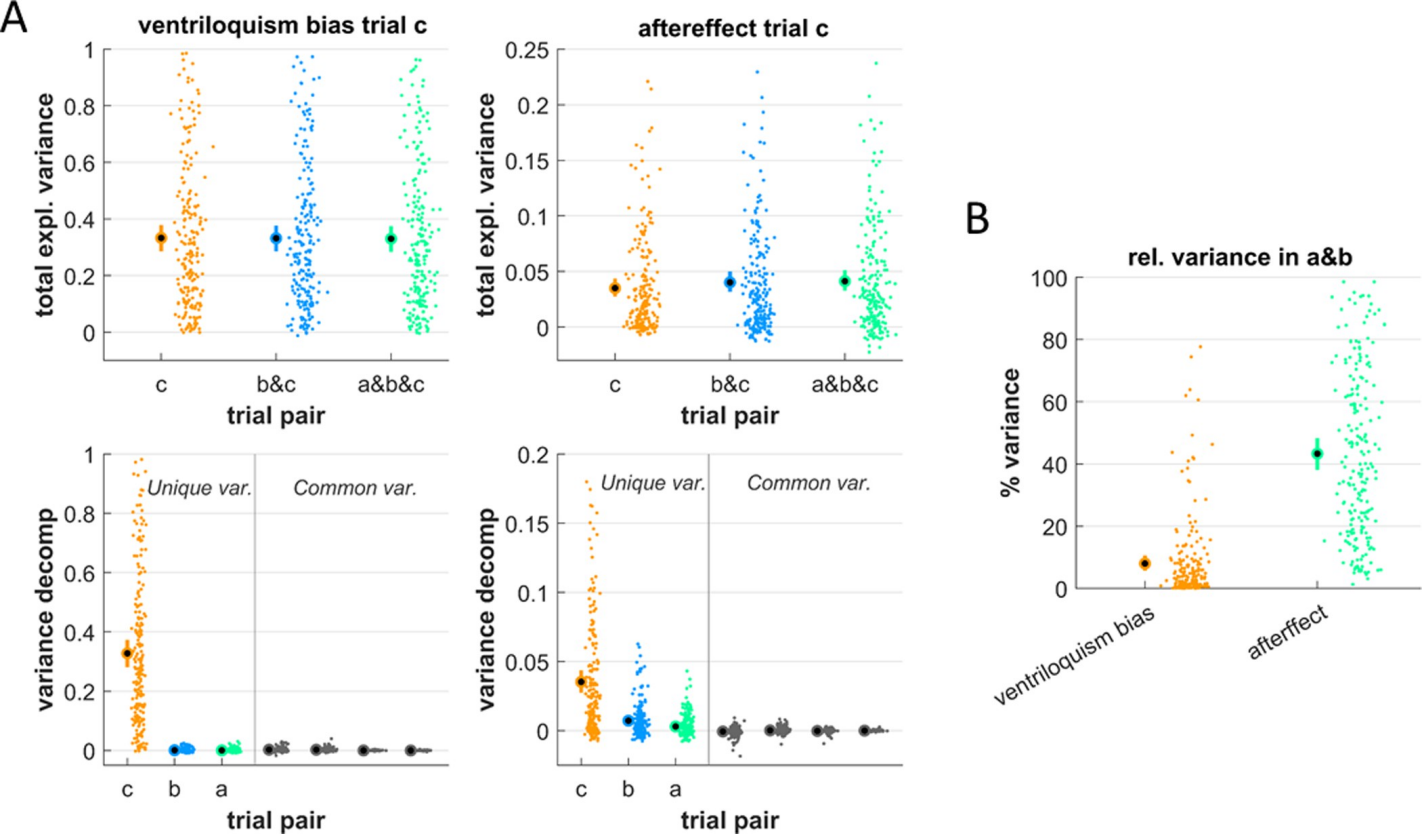
Modelling each bias against the spatial discrepancies in the immediate and preceding trial pairs. For each dataset we modelled the biases against the spatial discrepancy in the immediate AV trial-pair (here labelled c) and one or two preceding trial pairs (a,b). These models featured both linear and non-linear dependencies on each discrepancy to account for the nonlinearities visible in Fig 1. **A)** The upper panels display the total explained variance, the lower panels the results from a variance partitioning analysis. The latter assigns the explained variance to components uniquely explained by individual trials (in color) or shared variance that is jointly explained by different trials. Here the common variances were all negligible with mean values close to zero and/or 99% CIs reaching zero (from left to right: c and b, c and a, b and a, all three). **B)** Proportion of variance added by preceding trials. This was obtained for each dataset as the difference between the model relying on trials a&b&c and the model relying only on trial c, expressed relative to the variance for trial c in units of percent. Dots reflect individual datasets (i.e. participants, n = 205), circles the overall mean and error bars the 99% bootstrap confidence interval.

Hence for a more complete analysis we included all AV-A trial pairs and modelled the participant-wise biases based on the specific values of the spatial discrepancies in the immediate trial-pair and the two preceding trial-pairs. These models allowed for potential non-linear dependencies of each bias on the discrepancies. Fig 3 shows two results of this analysis. The upper panels in Fig 3A show the total explained variance for each bias when including only the immediate, one or two preceding trial-pairs. For the ventriloquism bias the explained variance was very similar for the three models, while for the aftereffect the explained variance increased when including previous trial-pairs. Indeed, the relative contribution of explained variance when ignoring the two preceding trial-pairs was less than ten percent for the ventriloquism bias (comparing model a&b&c to c only: 7.99% [5.75 10.65], mean and CI; Fig 3B), while it was more than five times this for the aftereffect (43.30% [38.38 48.14]). A direct comparison provided very strong evidence that the explanatory power provided by preceding trial-pairs was larger for the aftereffect (paired t-test: Cohens’ D = 1.66, BF = 10^40^).

In addition, we partitioned the explained variance into contributions uniquely explained by individual trial-pairs and the variance shared by these (Fig 3A, lower panels). The shared contributions were negligible, as expected given the largely independent values of spatial discrepancies across trials in this experimental design. For both biases the unique contribution of the immediate discrepancy was the strongest predictor (ventriloquism bias: 0.328 [0.283 0.374]; aftereffect: 0.035 [0.027 0.044]). Importantly, the contributions of the preceding trials differed between biases: these were negligible for the ventriloquism bias (trial-pair b: 0.001 [0 0.002]; trial-pair c: 0.000 [-0.001 0.001]) but not for the aftereffect (trial-pair b: 0.007 [0.005 0.010]; trial-pair b: 0.003 [0.002 0.005]), although the overall explained variance was larger for the ventriloquism bias. A direct comparison provided very strong evidence that the unique variance explained by the preceding trial-pairs was larger for the aftereffect (trial-pair b: Cohens’ D = 0.64, BF = 10^7^, trial c: D = 0.39, BF = 162). Hence, only for the aftereffect did the spatial discrepancy in preceding trial-pairs contribute significant explained variance.

In a third analysis we used linear mixed effects models to predict each bias across the entire sample of datasets based on linear and nonlinear dependencies on the discrepancies across up to three trial-pairs. Again we compared the predictive power of a model including only the immediate trial-pair (c) with models including one (b&c) or two preceding trial-pairs (a&b&c). For the ventriloquism bias the data revealed very strong evidence that they were best explained by a model not including preceding trial-pairs (relative BIC: 0, 26.1 and 105.9), while for the aftereffect we found very strong evidence that the inclusion of a preceding trial-pair provided a better account (relative BIC: 77.5, 0 and 35.8). This corroborates the above results that the ventriloquism bias is tied only to the immediate multisensory discrepancy while the aftereffect is shaped also by those in preceding trials.

### No contribution of the aftereffect to subsequent integration

The first analysis above shows that numerically both response biases may increase following a series of trials with a common direction of spatial discrepancy, though the increase was much more pronounced for the aftereffect. While this aftereffect is typically measured in unisensory trials, it could also shape responses during subsequent multisensory trials and thereby contribute to the numerically observed dependency of the ventriloquism bias on preceding discrepancies. That is, the aftereffect induced by (and measured in) one AV-A pair could shape the ventriloquism bias in a following AV trial. To probe whether this is the case, we implemented a more extended model for the ventriloquism bias that, in addition to the spatial discrepancies, also included the aftereffects in preceding trial-pairs. Specifically, we modelled the ventriloquism bias in trial-pair d based on the discrepancies in pairs b&c, and d and on the aftereffect in pairs a,b and c. We then used variance partitioning to probe whether the aftereffect contributes uniquely to shaping subsequent ventriloquism biases and whether it contributes common variance that overlaps with that explained by the spatial discrepancies. Overlapping variance could arise if an apparent history dependence of the bias is shaped by the preceding aftereffects. Unique variance would point to an influence of the aftereffect that is separate from the multisensory discrepancy, for example if the aftereffect constitutes a genuine modality-specific bias.

**Fig 4:**
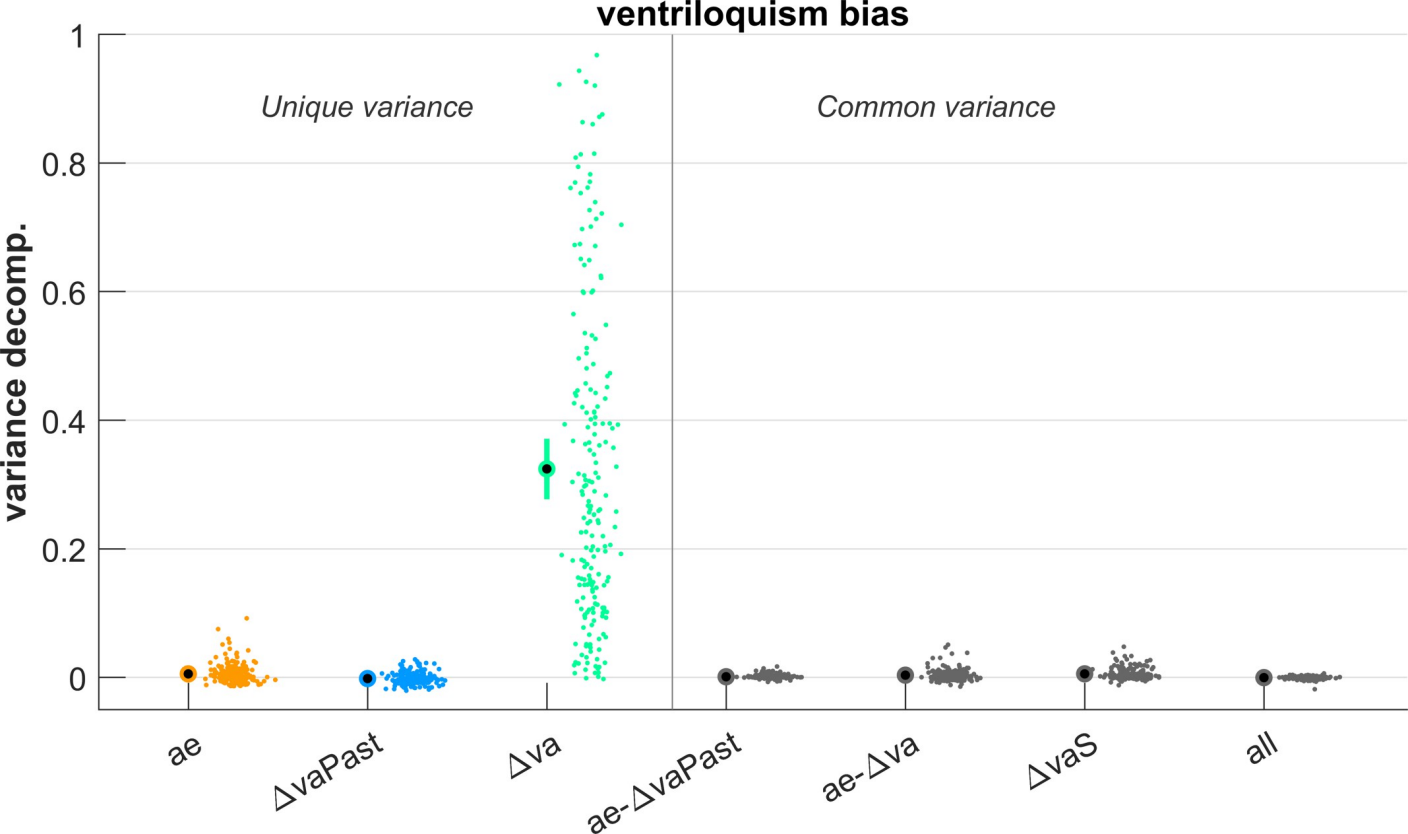
Modelling the ventriloquism bias against spatial discrepancies and previous aftereffects. We modelled the ventriloquism bias against the spatial discrepancy in the immediate AV trial-pair (labelled Δva) and two preceding trial pairs (labelled ΔvaPast). In addition, we included the aftereffect in three preceding trial pairs (labelled ae). These models featured both linear and non-linear dependencies on discrepancy and a linear dependency on the aftereffect. The graph shows the results from a variance partitioning analysis, with unique variances in colour and common variances in grey (here ae-ΔvaPast/ae-Δva are the variances shared by the aftereffect and the preceding / immediate discrepancies, ΔvaS denotes the variance shared by immediate and preceding discrepancies and all refers to the variance common to all predictor groups). Common variances were all negligible with mean values close to zero and/or 99% CIs reaching zero. Dots reflect individual datasets (i.e. participants, n = 205), circles the overall mean and error bars the 99% bootstrap confidence interval.

The results suggest that the aftereffect did not contribute significant unique or shared variance to the ventriloquism bias (Fig 4). As expected from the above, the immediate spatial discrepancy (termed ‘Δva’) explained a considerable variance (0.324 [0.280 0.370]), while the contribution of the preceding discrepancies was negligible (‘ΔvaPast’: -0.002 [-0.003–0.000]). Importantly, the unique contribution of the aftereffect was only marginal (‘ae’: 0.005 [0.003 0.008]) as was its common contribution with the immediate or preceding discrepancies (0.001 [0 0.002], and 0.003 [0.002 0.005]). Hence, any residual dependency of the ventriloquism bias that is reflected in these datasets is only marginally explained by specific contributions of previous spatial arrangements or the aftereffect.

## Discussion

Multisensory integration and recalibration are two phenomena by which perception deals with discrepant signals from our environment. Such discrepancies can be dynamic and may feature predictable and unpredictable components. Our results show that the spatial ventriloquism bias ‐ reflecting integration ‐ is shaped almost exclusively by the immediate multisensory discrepancy and not by those presented in preceding trials. In contrast, the ventriloquism aftereffect ‐ reflecting recalibration ‐ scales in a cumulative manner with these discrepancies. Hence these two biases exhibit distinct dependencies on the recent history of multisensory experience, supporting the idea that the two processes reflect distinct functional purposes.

### Multisensory integration as adaptive process

Our data support the notion that multisensory integration reduces discrepant sensory estimates between seemingly redundant signals [24, 27, 40]. In the large data sample analyzed here, the ventriloquism bias was driven mostly by the audio-visual discrepancy experienced in the current trial, while those experienced in the preceding multisensory trials had only a minimal influence, at best. Still, this result does not rule out that the overall integration process also reflects the history of the sensory environment. Integration scales with the relative unisensory reliabilities [41, 42] and our brain keeps track of these in a leaky manner [43]. This renders the integration weights assigned to the individual signals history dependent. Similarly, our brain tracks the presumed causal relations between multisensory signals, and the momentary estimate of whether two signals are deemed causally related depends on the recent exposure to discrepant stimuli [44, 45]. This history dependence of a common-cause prior provides a route for the past multisensory experience to enter the general integration process, similar to generic Bayesian models of perception in which the previous trial’s posterior influences the next trials prior [46].

However, our data show that previous multisensory discrepancies do not directly shape the integration bias. That is, the sign or magnitude of previously experienced discrepancies have very limited direct predictive power for the subsequent integration bias. This conclusion is in line with a previous study that was based on a smaller sample and relied on an experimental design featuring only one magnitude of spatial discrepancy [1]. In that study, the audio-visual stimuli were either discrepant or co-localized, which may influence the manner in which multisensory causal relations are estimated. In contrast, in the present experiments both the degree and direction of spatial discrepancies were unpredictable, demonstrating the lack of history-dependence of the integration bias in a more variable and possibly more natural context.

One could argue that any influence of previous trials on integration emerges in the form of spatial recalibration, whereby the previous trial-pair shifts auditory representations that bias the integration in subsequent AV trials. To test for such a propagation of auditory recalibration across trial-pairs, we probed the predictive power of the aftereffect bias on the subsequent ventriloquism bias. However, this contribution was numerically marginal compared to the multisensory discrepancy in the AV trial of interest, suggesting that contributions of cumulative recalibration to the ventriloquism bias are also negligible.

Across the three types of data analyses presented here, the numeric evidence for a history dependence of integration differed. The mixed effect model across all datasets was clearly in favor of no history-dependence and the participant-wise models attributed negligible unique variance to previous trials. This suggests that the slight numeric differences in the ventriloquism bias between sequences of trial-pairs with same or changing discrepancies in Fig 2 possibly result from comparing the specific selected trials and does not generalize to the full datasets as analyzed using linear models.

### Multisensory recalibration accumulates over time

Our results show a clear cumulative effect of preceding multisensory discrepancies on the aftereffect. This observation is in line with the known adaptive nature of the aftereffect, which is classically probed following several minutes of exposure to discrepant audio-visual stimuli [2, 4, 11, 47–49]. However, recent studies have shown that this aftereffect emerges also on a trial-wise basis [1, 5, 6] and some have shown that the aftereffect accumulates over a series of up to 5 [6, 9] or twenty trials [1, 8] or a few minutes [10].

Our results extend those studies in a number of ways. First, most studies probing the influence of immediately preceding trials relied on a fixed magnitude of the spatial discrepancy, or even a fixed sign [1, 9]. Such a high predictability may foster the cumulative nature of the aftereffect and may engage additional learning mechanisms not present in more naturalistic environments involving pseudo-random and unpredictable discrepancies as used here. Second, the locations of the auditory stimuli in AV and subsequent A trials were either the same or limited to within a 10° range in previous work [1, 9]. In contrast, in the present experiments they varied to a much larger extend, requiring recalibration to generalize over a larger proportion of the auditory space. Hence, the simple repetition of previous judgements that participants may have used in previous studies was not possible in this design. Third, because of their design, these studies did not directly quantify the independent contribution of the preceding trials, which the variance decomposition here does. Furthermore, eye movements or the direction of fixations may confound trial-wise recalibration [5, 50, 51], which emerges in a mixture of head- and eye-centered reference frames [52]. To avoid such confounding effects, the present study relied on very short auditory and visual signals, making it unlikely that participants moved their eyes during stimulus presentation (see also discussions in [1, 5, 16]).

### How are integration and recalibration linked?

One view stipulates that recalibration is driven by the discrepancy between integrated multisensory signals and a subsequent unisensory stimulus, rendering recalibration directly dependent on the outcome of integration [11, 13, 17]. If two multisensory signals are deemed sufficiently discrepant to unlikely originate from a common source, the outcome of multisensory causal inference should emphasize the task-relevant unisensory estimate, leaving no multisensory bias to drive recalibration. Hence, in this view integration becomes a prerequisite for recalibration similar to development where integration seems to emerge prior to recalibration [12]. While our data cannot rule out that integration per se is required for recalibration to emerge, our data speak against the hypothesis that both processes are directly linked by a similar dependency on preceding multisensory discrepancies.

An alternative view holds that recalibration is shaped by the believe in a modality-specific bias, hence a bias that in the present setting pertains to only the auditory system [13, 17–20]. This belief may be shaped by multiple factors, including judgements about the causal relation of sensory signals as one of many factors. As a result, both integration and recalibration tend to correlate across experimental manipulations and the immediate multisensory discrepancy. However, a residual ventriloquism aftereffect emerges also when auditory and visual signals are not judged as originating from a common source [6], when obviously not originating from a common location [47] and when attention is directed towards task-unrelated visual stimuli [53]. Hence, recalibration is not directly contingent on multisensory signals to be judged as relating to the same object. Our results support this view and speak in favor of distinct functional roles of integration and recalibration that are shaped by the immediate multisensory discrepancy as just one of many factors.

The collective evidence is in line with a model of Bayesian causal inference that shapes multisensory perception in general, but which affects integration and recalibration via distinct mechanisms [13]. Unpredictable discrepancies between auditory and visual stimuli are reduced by multisensory integration, and the brain continuously updates the a priori belief in a common cause of multisensory signals based on the recently experienced discrepancies [25, 28, 43]. At the same time, the representation of auditory space is continuously updated in a leaky manner to compensate for short-term changes in audio-visual disparities and to minimize apparent localization errors [21, 52]. The respectively underlying estimates of audio-visual disparities may be indirectly shaped by the common cause prior, but it is possible that distinct time scales of this are relevant for integration and recalibration. In particular, for integration that primarily depends on the instantaneous bimodal signals, the common cause evidence should be based on a short time scale, whereas for recalibration that depends on a series of bimodal signals it should be based on a longer time scale. Testing this prediction may not be easily feasible with the very brief stimuli used here, but for example paradigms involving audio-visual motion or visuo-motor paradigms involving hand movements and manipulated visual feedback could allow this [54, 55].

## Limitations of the study

We note that there are several features of the present experiments that constrain the interpretation of the results. First, similar to most previous studies the present experiments relied on a predictable trial structure in which AV trials were always followed by A trials. This repetitive pattern may strengthen the influence of preceding multisensory discrepancies, as the occurrence of any multisensory stimulus (versus a unisensory stimulus) in the trial sequence can be predicted. However, this feature is unlikely to solely explain the differential history dependence of the ventriloquism bias and the aftereffect, given that a cumulative effect of experienced discrepancies on the aftereffect has also been observed with pseudo-randomized sequences of trials [6].

Second, the stimuli used here featured multisensory discrepancies that varied randomly between trials and which were distributed over a range of about ±25° for some and up to about ±50° for other experiments. While this range is similar to other typical studies on the ventriloquism effect, the range of experienced discrepancies can shape the overall evidence for a causal relation of the auditory and visual stimuli [22]. When the range of experienced discrepancies is very large, the sensory evidence for a common cause of the specific visual and auditory stimuli experienced in a given trial may be very high in some and low in other trials. In contrast, for studies involving a fixed discrepancy this evidence is constant. As a result, the a priori binding tendencies for participants in the two lines of studies will differ and this may alter the degree to which cumulative discrepancies affect integration or recalibration. In fact, in a previous study we found that the context of experienced discrepancies affects integration and recalibration differently [22], which may explain some of the differences between studies relying on small, fixed or very predictable discrepancies and studies in which the presentation of multisensory stimuli is distributed and randomized over a considerable portion of the visual field.

Third, the present paradigms relied on visually-guided judgements whereby participants moved a visual cursor to the perceived location of a sound. This effectively constitutes a crossmodal judgement of the sound position, in which the sensory evidence to-be-judged and the sensory evidence used to actually make the judgment are not in the same modality. This is in contrast to for example the few visual trials (not analyzed here), where the sensory evidence to-be-judged and the sensory evidence used to make the judgement (cursor) are in the same modality. To our knowledge little is known about how the nature of the judgement modality affects the biases observed in the ventriloquism paradigm. Given that other studies have also relied on other response modes such as pointing, the overall emergence of the ventriloquism bias is robust [56]. Data from studies in which participants relate visual and proprioceptive evidence, however, suggest that the reliability of intramodal judgements is higher compared to crossmodal judgements [57–60] and this differential reliability also shapes the outcome of multisensory integration [54]. Generalizing to the present paradigm, we conclude that the crossmodal nature of the relevant judgments may result in a particularly strong integration bias, likely enhancing the sensitivity of this bias to various sorts of influences. If anything, this should enhance any history dependence of ventriloquism bias. It is hence unlikely that the present results are specific to the type of judgement used here, but future work is required to understand how the nature of the response modality shapes multisensory bias in the ventriloquism paradigm.

## Supporting information

S1 File The supporting information contains methods details for analysis #2 and analysis #3. It also contains a table with the model parameters for the analysis in Fig 3.
(DOCX)
