# Supplementary material for: Cumulative multisensory discrepancies shape the ventriloquism aftereffect but not the ventriloquism bias
Source: PLoS One. 2023 Aug 22;18(8):e0290461. doi: 10.1371/journal.pone.0290461 (PMC10443876; doi:10.1371/journal.pone.0290461)
Supplement: S1 File — It also contains a table with the model parameters for the analysis in Fig 3. (DOCX) [file pone.0290461.s001.docx]

**Supplemental details for the second analysis (relating to Figure 3)**

Results of a regression analysis predicting the participant-wise bias (in trial-pair c) based on the spatial discrepancies in the immediate trial (c) and previous trial-pairs (trials a and b). For each participant and bias we fit the following model across all trials

*bias* ~ 1 + Δ_c_va + Δ_c_va^0.5^ + Δ_b_va + Δ_b_va^0.5^ + Δ_a_va + Δ_a_va^0.5^

The tables below list the weights for the spatial discrepancies and the results of a significance test against zero (two-sided t-tests). The explained variance of the predictors relating to trials a,b,c are shown in Figure 3.

|  | Δ_a_va | Δ_b_va | Δ_c_va | Δ_a_va^0.5^ | Δ_b_va^0.5^ | Δ_c_va^0.5^ |
| --- | --- | --- | --- | --- | --- | --- |
| b | 0.007 | -0.002 | 0.004 | -0.011 | 0.067 | 1.387 |
| t | 0.878 | -0.255 | 0.167 | -0.274 | 1.774 | 17.523 |
| p | 0.3811 | 0.799 | 0.8672 | 0.785 | 0.0775 | <10^-5^ |
| CI (95%) | [-0.009 0.023] | [-0.017 0.013 | [-0.047 0.056] | [-0.089 0.067 | [-0.0008 0.142] | [1.231 1.543] |

**Table 1: Results for the ventriloquism bias.**

|  | Δ_a_va | Δ_b_va | Δ_c_va | Δ_a_va^0.5^ | Δ_b_va^0.5^ | Δ_c_va^0.5^ |
| --- | --- | --- | --- | --- | --- | --- |
| b | 0.012 | 0.010 | 0.032 | -0.004 | 0.032 | 0.076 |
| t | 1.654 | 1.130 | 3.516 | -0.107 | 0.767 | 1.810 |
| p | 0.0992 | 0.2600 | 0.0005 | 0.9152 | 0.4437 | 0.0718 |
| CI (95%) | [-0.002 0.027] | [-0.008 0.028] | [0.014 0.050] | [-0.074 0.067] | [-0.050 0.113] | [-0.007 0.159] |

**Table 2: Results for the aftereffect.**

**Supplemental details for the third analysis**

In this analysis we fit the collective data across all experiments using linear mixed effect models. For each bias we fit a model including only a dependency on the discrepancy in trial c, a model also including trial b and a model including all three trials. We then compared the predictive power of these models using their BIC values.

*bias* ~ Δ_c_va + Δ_c_va^0.5^+ (1|Subj) + (1|Exp) + (Δ_c_va |Exp) + (Δ_c_va^0.5^|Exp)

*bias* ~ Δ_c_va + Δ_c_va^0.5^+ Δ_b_va + Δ_b_va^0.5^+ (1|Subj) + (1|Exp) + (Δ_c_va |Exp) + (Δ_c_va^0.5^|Exp)+ (Δ_b_va |Exp) + (Δ_b_va^0.5^|Exp)

*bias* ~ Δ_c_va + Δ_c_va^0.5^+ Δ_b_va + Δ_b_va^0.5^+ Δ_a_va + Δ_a_va^0.5^ + (1|Subj) + (1|Exp) + (Δ_c_va |Exp) + (Δ_c_va^0.5^|Exp)+ (Δ_b_va |Exp) + (Δ_b_va^0.5^|Exp) + (Δ_a_va |Exp) + (Δ_a_va^0.5^|Exp)
